# A high-precision hierarchical registration approach for stain- and scanner-independent colocalization on whole slide images in histopathology

**DOI:** 10.1007/s13755-025-00353-7

**Published:** 2025-05-23

**Authors:** Tom Bisson, Michael Franz, Tim-Rasmus Kiehl, Peter Boor, Peter Hufnagl, Norman Zerbe

**Affiliations:** 1 Charité – Universitätsmedizin Berlin, corporate member of Freie Universität Berlin and Humboldt-Universität zu Berlin, Institute of Medical Informatics, Invalidenstraße 90, 10115 Berlin, Germany; 2 Charité – Universitätsmedizin Berlin, corporate member of Freie Universität Berlin and Humboldt-Universität zu Berlin, Institute of Pathology, Virchowweg 15, 10117 Berlin, Germany; 3 Berlin Institute of Health at Charité – Universitätsmedizin Berlin, Charitéplatz 1, 10117 Berlin, Germany; 4 University Clinic Aachen, RWTH University, Aachen, Germany; 5 EMPAIA International e.V., Berlin, Germany

**Keywords:** Image registration, Colocalization, Histopathology, Whole slide imaging

## Abstract

**Purpose:**

The paper presents a high-precision hierarchical registration method to accurately align image coordinates across Whole Slide Images of histopathological slides. The proposed technique was designed to achieve robust and pixel-precise stain- and scanner-independent colocalization. It addresses well-known challenges of histopathological imaging and differences arising from various staining protocols and digitization processes.

**Methods:**

Our method leverages the Elastix registration framework to achieve exceptionally precise colocalization of cell nuclei and other similarly sized tissue structures. By utilizing the pyramidal data structure of Whole Slide Images, we developed a hierarchical, multi-stage registration algorithm in which the transformation is gradually refined from a macroscopic to a microscopic scale.

**Results:**

Unlike other work in the field, our approach focuses on the colocalization of tissue structures rather than the alignment of the image data. The algorithm achieved sub-micrometer accuracy in colocalization, outperforming state-of-the-art solutions. We propose two distinct registration strategies to minimize the computation time, considering the spatial distribution of the coordinates.

**Conclusion:**

This algorithm is designed exclusively to compute point cloud transformations within Whole Slide Images, achieving a high accuracy at the expense of computation time. Consequently, it should be considered a highly specialized solution tailored to a specific subset of the registration problems occurring in digital pathology.

## Introduction

### Motivation

In computer science, image registration is an iterative process to arrive at a geometric transformation that provides the best alignment of one or more images to a reference image. In medical imaging, the need for image alignment arose from the requirement to examine temporal or spatial alterations in anatomical structures visually or to allow comparison of similar objects from different modalities or specimens. While in radiological images, excessive deviations between the individual image contents are primarily caused by physiological processes, patient movements, or the imaging system itself, the reasons for alignment and content differences in histopathological images are partly different. High-resolution scans of histological images, prepared from fixed tissue of biopsies or resections, are generally used in this context. These digitized slides may differ, for instance, in terms of tissue type, staining, focus, or scanning device. Furthermore, preparation and digitization can both introduce artifacts that must be intercepted during registration. In addition to a more sophisticated understanding of the three-dimensional orientation of tissue structures, the registration of slide sections for the colocalization of various biomarkers is an essential tool.

### Challenges

For image registration, robust algorithms and toolboxes have been developed to solve more complex problems, such as missing object fragments, wrapped and overlapping structures, or strongly diverging intensity distributions. However, most of these problems are complex and cannot be solved with a linear, out-of-the-box approach. Depending on the dataset characteristics, a tailored approach is required for each use case. For example, a set of parameters may be successful for a particular dataset but fail completely for another, slightly different one. Therefore, registration methods previously developed cannot be applied to image data in digital pathology without some adaptation. High-resolution scans of histological sections, so-called Whole Slide Images (WSIs), commonly have resolutions of around 0.25 μm per pixel, which causes a 1 cm^2^ piece of tissue to require about 4.5 GB of memory. If a WSI contains a particularly large resection or several large pieces of tissue, it easily reaches file sizes that exceed the usual memory capacities in personal computers or workstations. However, even with smaller file sizes, fast and efficient image data processing is only possible to a limited extent. One approach to working with such high-resolution images is to store the image in different resolution levels and to additionally tessellate these individual levels, i.e., to split the image into several tiles to enable fast and targeted access to relevant image sections. However, there are also particular challenges concerning the image content. These include the fact that the images to be registered can differ significantly in terms of staining, saturation, and intensity. Even if the two slides are standard hematoxylin and eosin (H&E) stains, they may differ considerably depending on the respective laboratory. The differences will be even more significant if the slides come in different tinctorial stains. The scanners and respective scanning profiles used in each case also significantly affect the appearance of the WSIs. Furthermore, variations arise during the preparation of the histological sections, such as different orientations on the slide or damage to the tissue, including tears, folds, or overlaps, introducing additional challenges during registration.

### Related work

A significant achievement for the registration of medical image data has been the Elastix framework [1], which provides a modularized and highly configurable toolbox for rigid and non-rigid registration based on the ITK framework [2]. Although its main focus is on radiology, the framework is intended for use in any imaging modality and also beyond the scope of medical imaging. The registration of WSIs is particularly challenging, as image sizes of more than 100,000 × 100,000 pixels are common. Due to these large dimensions, Elastix cannot be used directly to perform image registration on higher resolution levels. A common approach is to use downsampled versions of the WSI, which may already be sufficient for many applications. Moreover, it is not always necessary to use the entire tissue area. In a hierarchical procedure, a coarse global registration at low resolution can be performed first, followed by the registration of smaller patches at a higher resolution, using the gathered co-localized information from the higher hierarchical level. This way, Elastix could already be used on WSIs of consecutive histologic slides to co-localize tissue structures [3, 4] and to perform 3D reconstruction [5]. Furthermore, pathological and radiological image data could be correlated by multi-sensor registration of WSIs on MRI images [6, 7]. In addition to the primarily intensity-based registration methods, feature-based techniques have become quite popular in pathology. For instance, consecutive sections with different stains have been successfully registered using the patented SIFT feature descriptor [8]. Other descriptors, some of them open source, such as ORB, SURF and KAZE, were also able to demonstrate their capabilities [8]. However, maximizing accuracy has not been the primary focus in all cases. In clinical routine diagnostic work, there are applications where fast digital processing of histologic image data is critical. For example, Mueller et al. developed a time-efficient method that allows parallel viewing of consecutive tissue sections based on a single global registration [9]. Pixel-precise registration is not even necessary here since pathologists will never be pixel-precise when switching between individual WSIs and will have to reorient either way. For precise gigapixel-wise registrations of WSIs, Chandler et al. [10] presented a novel pipeline that iteratively aligns and rewrites WSI images in the Open Microscopy Environment (OMERO) file format. Furthermore, Wodzinski et al. [11] implemented a pluggable framework operating on larger image dimensions. However, it is important to note that the primary focus is on the comprehensive transformation of WSIs, and the tools are primarily designed for visualization purposes. 

### Contributions

The classical approach to image registration results in one or more sets of transformation parameters that can be used to map one image to another entirely. In pathology, however, it is not always practical to fully transform WSI tissue sections. This is partly because distortions, tissue defects or larger gaps between tissue fragments can occur during the various histological processing steps. Thus, when two consecutive sections are taken from the same tissue block or when the same section is stained and scanned multiple times, the images may not necessarily be congruently superimposable. If the transformation parameters are then optimized regardless, a loss of precision may occur in the areas that actually coincide. Although this loss is minimized in the optimization phase, it cannot be eliminated entirely. Consequently, instead of following the classical registration approach and transforming large regions or even the entire tissue, we follow the co-localization approach for re-stained and re-scanned tissue slides, where the corresponding structures on the images to be registered are mapped to each other individually. Our approach, therefore, aims at maximum accuracy, which is again achieved at the expense of computational time. These correspondences can then be further used depending on the specific problem to be solved.

## Materials and methods

### Data

The dataset used to develop the registration algorithm includes over 200 histological specimens from a breast cancer cohort. All slides were initially stained with standard H&E and digitized using five different scanners from 3DHISTECH (Budapest, Hungary), Hamamatsu Photonics (Hamamatsu, Japan) and Leica Biosystems (Wetzlar, Germany) at three different research centers. A 3DHistech P1000 scanner was used to acquire WSIs at 80× magnification, while the other four devices were used for scans at 40x. Subsequently, these slides were re-stained with an immunohistochemical (IHC) stain for phospho-histone H3 (PHH3) and re-scanned with the P1000 at 80x.

**Fig. 1:**
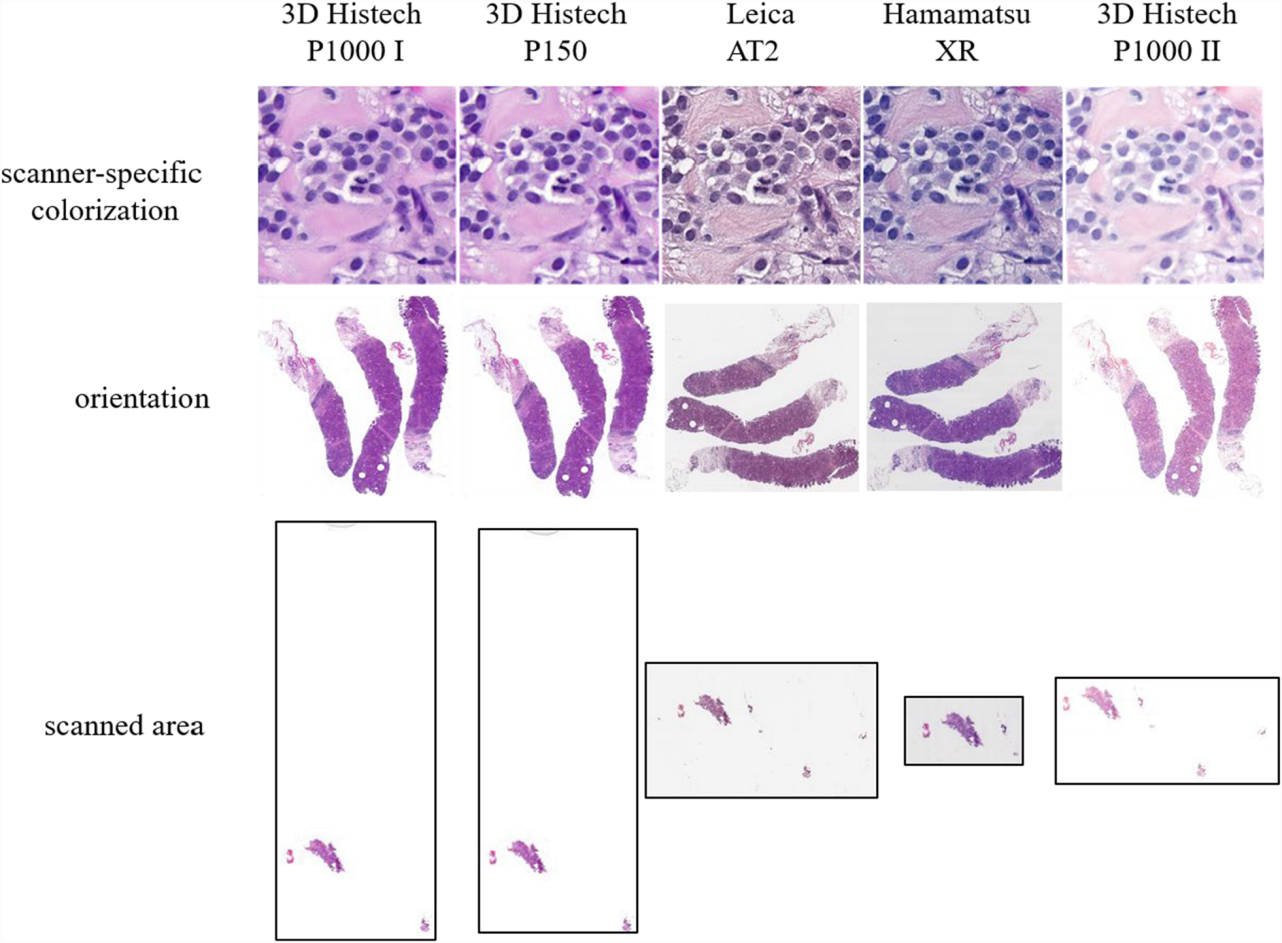
Varying WSI appearance between five scanners, depending on the specific device and manufacturer

There are several known challenges when working with WSIs. Guerrero et al. [12] have subdivided these into three categories: biological variation, technological variation, and pathological variation. Of these, the technical aspects are particularly important for image registration of histopathological slide sections. These tend to occur during the preparation of histological sections, i.e., artifacts such as tissue folds, tears, or even differences in staining intensities, and are further exacerbated in the image acquisition process. Another class of problems arises in the context of digitization, where, for example, blurring, illumination, and contrast problems may arise [13]. Beyond these obvious and identifiable artifacts, there may be other digitization problems that only become relevant when working with images from different WSI scanners. Indeed, there can be significant differences between WSIs generated from the same histological slide but using different scanners. Three of the main differences between the scanners are shown in Fig. 1.

Depending on the scanner, the visual appearance of WSIs can vary significantly in intensity and coloration. Since these differences are not equally prominent for all tissue structures present, the possibilities of intensity-based registration are already limited. Given that scanners of different manufacturers produce WSIs with varying base coordinate systems and initial orientations, it is a crucial step to address this initial displacement prior to the actual registration process to avoid unnecessarily complicating the optimization of the transformation. However, a more severe problem arises from the preprocessed, automated tissue segmentation of the scanner devices used to determine the area to be scanned. When scanning IHC sections, brighter areas with only a few to no darker signals (e.g., stained nuclei) may be incorrectly classified as background and therefore not scanned. This problem also occurs in HE slides, particularly in areas with a lot of fatty tissue, which have poor contrast to the background. Furthermore, scratches, dust, or other contamination can cause a larger area of the slide to be scanned. If this area differs between several WSIs of the same section, this must also be intercepted during registration.

Despite the same magnification on four of the five scanners, the resolution of the WSIs varies between scanners, with the largest difference being between the Hamamatsu XR at 0.2272 mpp (micrometer per pixel) and the 3DHISTECH P150 at 0.2744 mpp (see Table 1), resulting in structures appearing roughly 20% larger on the P150.

**Table 1:** Final WSI resolutions in the dataset

Scanner	Leica AT2	Hamamatsu XR	3DHISTECH P150	3DHISTECH P1000 I (40x)	3DHISTECH P1000 II (80x)
Resolution	0.2514 mpp	0.2272 mpp	0.2744 mpp	0.2454 mpp	0.1213 mpp

This shows that scaling for inter-scanner registration is inevitable, even when the same tissue is scanned at the same magnification level. On the scanned slide sections, 8334 mitoses were annotated in selected tumor regions on H&E WSIs (scanned by a 3DHISTECH P1000 with 40× magnification) and 2533 mitosis on the PHH3 WSIs (scanned by a 3DHISTECH P1000 with 80× magnification).

**Fig. 2:**
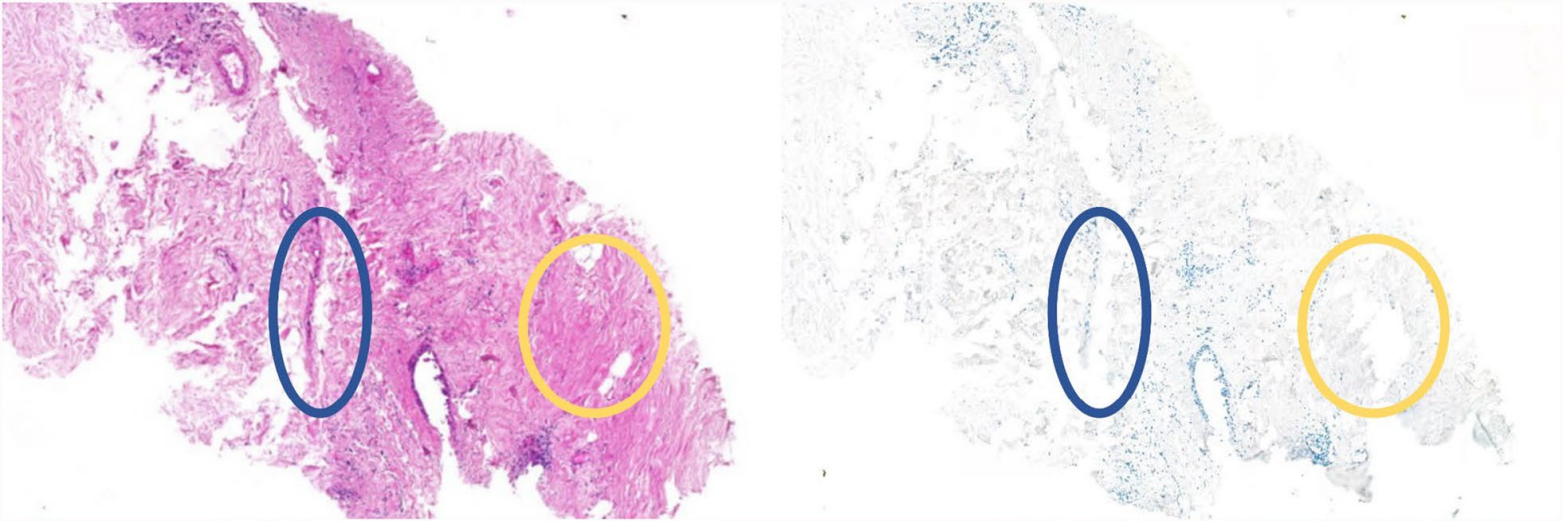
Minor tissue deformations occurring when the original H&E slide (left) is re-stained for PHH3 (right)

Much more striking morphological differences may occur between different stains. In standard H&E staining, for example, the cell nuclei are highlighted by the blueish hematoxylin. In IHC stains, hematoxylin is the usual counterstain to the brownish Diaminobenzidine (DAB) chromogen that labels the specific antigen of interest. Therefore, even when re-staining a slide, the DAB may bind beyond the edge of the nucleus and cause a change in the visual appearance of that same morphological structure. Furthermore, the process of re-staining a slide can lead to further deformations. In this process, antigen unmasking steps, such as microwaving or citric acid treatment, may cause part of the tissue to be washed off the slide. However, more frequently, minor deformations, such as tears or tissue displacement, resulting from the mechanical treatment of the slide are the consequence (see Fig. 2).

### Concept

Based on the available dataset and the observations made during the manual and automatic digitization of the slide sections, we searched for a novel registration method to generate valid point or annotation mappings from one coordinate system of a reference image I_F, called fixed image to another coordinate system of a corresponding image I_M, namely moving image. In this context, the base layer of a WSI represents the actual image data scanned with the maximum magnification. Since the image data on this base layer usually exceeds common image data sizes and thus usual memory capacities, we have to resort to a hierarchical tile-based approach, both for accessing the image data and for image registration and transformation. Reading and viewing WSIs is mostly implemented in a pyramid-shaped form containing different levels of resolution of the base image, usually sampled down as powers of two, as is done by most slide scanner vendors [14]. Most registration frameworks support multi-resolution strategies that process images from coarse to fine resolution. Still, these cannot be easily applied to images with the dimensions mentioned above, as this would consume considerable memory resources. However, we can take advantage of this strategy by combining the hierarchical approach with a tile-based method on the base layer or a respective level with a similar detailed resolution. This requires a multi-staged registration, starting with a coarse alignment of the whole tissue fragment on a downsampled magnification level (for instance, with a 5× magnification), resulting in an initial transformation that gives a good first estimated alignment of the depicted objects. Crucial for this step, however, is an initial segmentation and detection of the scanned region on which the specimen is located. As mentioned earlier, the actual scanned area of the slide can vary dramatically from scanner to scanner due to internal detection algorithms or configuration profiles. To compensate for these discrepancies, a pre-segmentation is applied before the coarse registration is performed. If the automated segmentation fails and the desired results are far off, the tooling should allow subsequent manual adjustment of the recognized image region. Since the area to be aligned with should be kept as small as possible, the region of interest (ROI) should be tightly bound to the relevant object’s shape. The overview images of these regions will then serve as input images for the initial coarse registration, whereby the respective resolution level is dependent on the magnification of the particular slide scans. This means that for a slide scanned at 80× magnification, an initial resolution level of 5, and thus a downsampling factor of 2^5^, would be reasonable. In contrast, for a corresponding slide scanned at 40× magnification, a resolution level of 4, and thus a downsampling factor of 2^4^ would be reasonable. Therefore, anatomical structures on the fixed image should roughly match the size of these structures on the moving image. This is achieved by a similarity transformation during registration. The resulting transformation parameters are then used to obtain corresponding image tiles on the base layer of our moving WSI and will be used in the subsequent registration step, which is necessary to further correct and refine the already computed transformations. While for most applications, either the correction or the refinement step is already sufficient to achieve a high degree of accuracy, in certain cases, it may be useful to perform a prior refinement step before computing the final correction parameters.

**Fig. 3:**
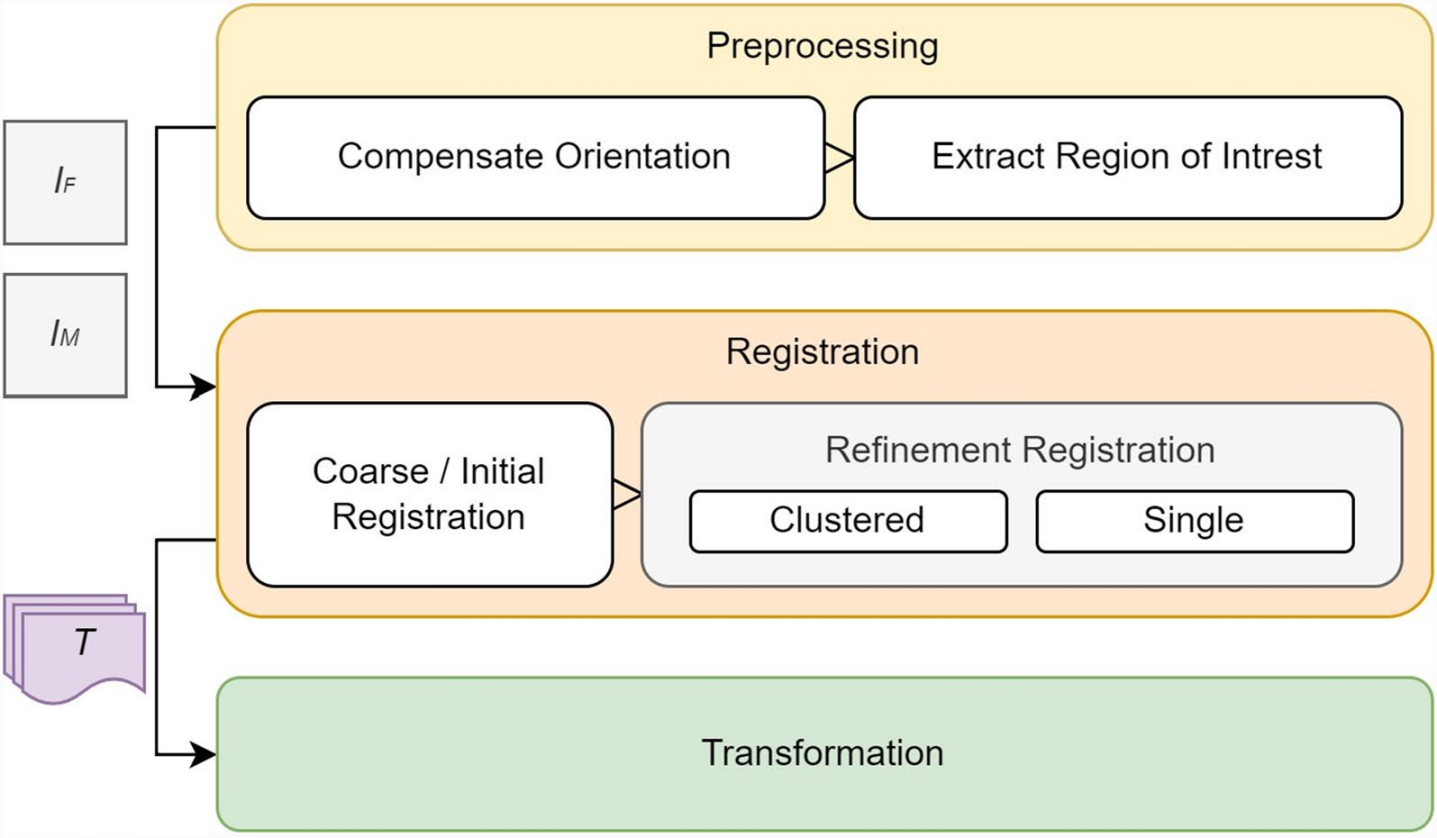
Overview of the general registration workflow. I_F and I_M mark the fixed and moving image, while T is the transformation parameter set mapping points from I_F to I_M

The whole process is outlined in Fig. 3 and described in the following sections. The process is subdivided into three major components, starting with preprocessing to harmonize the image data to a common technical and digital presentation. Moreover, the computation (registration) and application (transformation) of the resulting transformation parameters are separated. This allows logical separation and, thus, the application of registration and transformation independently of the underlying dataset. Thus, preprocessed transformation matrices can eventually be applied to an arbitrary point set in the fixed image domain. Since the core process is primarily aimed at pixel-wise registration, non-rigid registration techniques can be avoided in most cases, which only prolong the computation time and may give incorrect and discontinuous transformation results. The entire process is illustrated as pseudocode in Algorithm 1.

**Algorithm 1:**
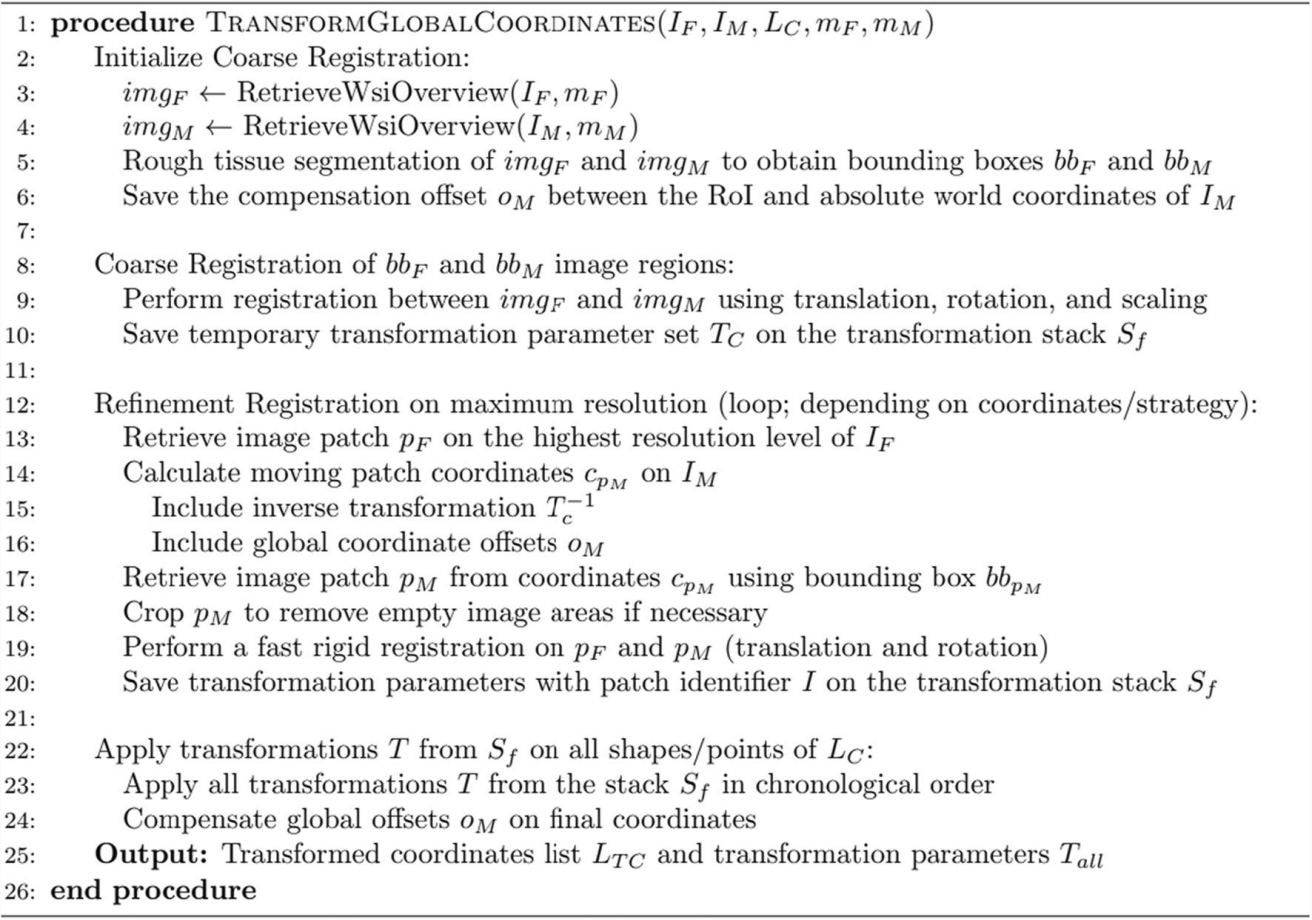
Pseudocode illustrates the global process of mapping image coordinates from one WSI to another

### Preprocessing

Several requirements that we defined in the previous sections, Data and Concept, must be met in advance to create a common internal representation to process WSI data independently of the origin, the characteristics or other inferences related to the underlying technical entity of the virtual slide or the tissue itself. This first includes a uniform interface to read image data and associated metadata from proprietary WSI files and provides a convenient way to access the algorithm. Since we depend on the support of certain proprietary file formats due to the heterogenous input data (e.g.,.svs for Leica Aperio GT450,.ndpi for Hamamatsu XR and.mrxs for 3DHistech P1000/P250), we initially opted for a closed-source library (Virtual Slide SDK by VMscope) instead of an open-source framework such as OpenSlide.^1^ Despite this limitation, the tool is decoupled to such an extent that the library for reading the image data can be exchanged without problems.

Despite having scanned the same tissue, it may differ in its initial orientation and presentation due to scanner-inherent processes, compensation for major differences between fixed and moving image domains is required. Through segmentation, the rough outlines of the objects to be aligned can be assessed to create an initial region of interest for the corresponding image slides. Pre-segmentation of the tissue is performed by a rather naive segmentation approach in which the relevant tissue is separated from the background using Otsu thresholding and morphological operations (e.g., dilation followed by erosion and filling of holes). The relevance of the individual contours, to be considered as a particle of interest, is determined by the mean size and its standard deviation as well as by its distance from the mass center of the contours, ensuring that most irrelevant small tissue fragments and artifacts on the scanned slide region will be excluded. Due to tissue alterations during the restaining process or generally distinct expressed tissue structures, the segmentation algorithm is not entirely robust and reliable, demanding manual corrections of the bounding boxes surrounding the relevant tissue in some cases. Additionally, significant orientation discrepancies, such as 90-degree rotations or vertical and horizontal flips of the WSI, as well as major magnification differences, need to be configured as application runtime parameters. All of these operations necessitate pre-transformation of the coordinates of the input annotations on the fixed image, accordingly.

### Registration

In the preliminary steps, the WSIs and related coordinates were processed in a way that allowed the registration of the actual tissue regions without having to deal with the particularities of the different scanners and image formats. The relevant image regions are defined as coordinates of a rectangle *Rec[roi_fixed]* and *Rec[roi_moving]* in the corresponding WSI coordinate system. The actual image data on a specific resolution level is then obtained by sampling down the rectangle coordinates and passing it to the data access interface.

The primary step is a rough and fast registration of the overview images at a reasonably low-resolution level that produces a good initial approximation of the transformation. Therefore, a simple rigid registration is sufficient at this step as the results will be refined in further iterations. The registration process itself relies on a multi-resolution strategy as well. The coarse resolution level is configurable and should be chosen depending on the size of the image data and the available memory. As there might still be a slight magnification offset due to different size interpretations and minor mechanical imprecisions of scanning devices, we perform the coarse registration based on a similarity transform model, compounded by a rigid body transformation and an isotropic scaling where the moving image is initialized at the geometrical center of the fixed image. The basic transformation is then defined by an equation that considers a scalar scaling vector, a 2D rotation matrix and a translation vector. By including the center of rotation in this equation, this results in a parameterization vector of size four [15]. To optimize the transformation of the moving image, an adaptive stochastic gradient descent approach (ASGD) is used, a robust and fast optimization method [16]. A variant of Mutual Information (MI) by Mattes is applied for similarity measure, utilizing the Parzen Windows technique to estimate continuous histogram densities with a random sampling strategy [17]. MI is a useful metric when dealing with images from different modalities, in our case, different stains, as it measures similarity based on the occurrences of intensity probabilities and not on actual intensity values. To prevent the mapping to non-pixel coordinates, a BSpline-interpolation technique is used, ensuring a high accuracy grade of pixel interpolation. The same methods are used for the subsequent refinement step, only deviating in the specific parametrization. The default parameters can be overwritten anytime by defining an external parameter file and stating it in the application’s configuration.

**Fig. 4:**
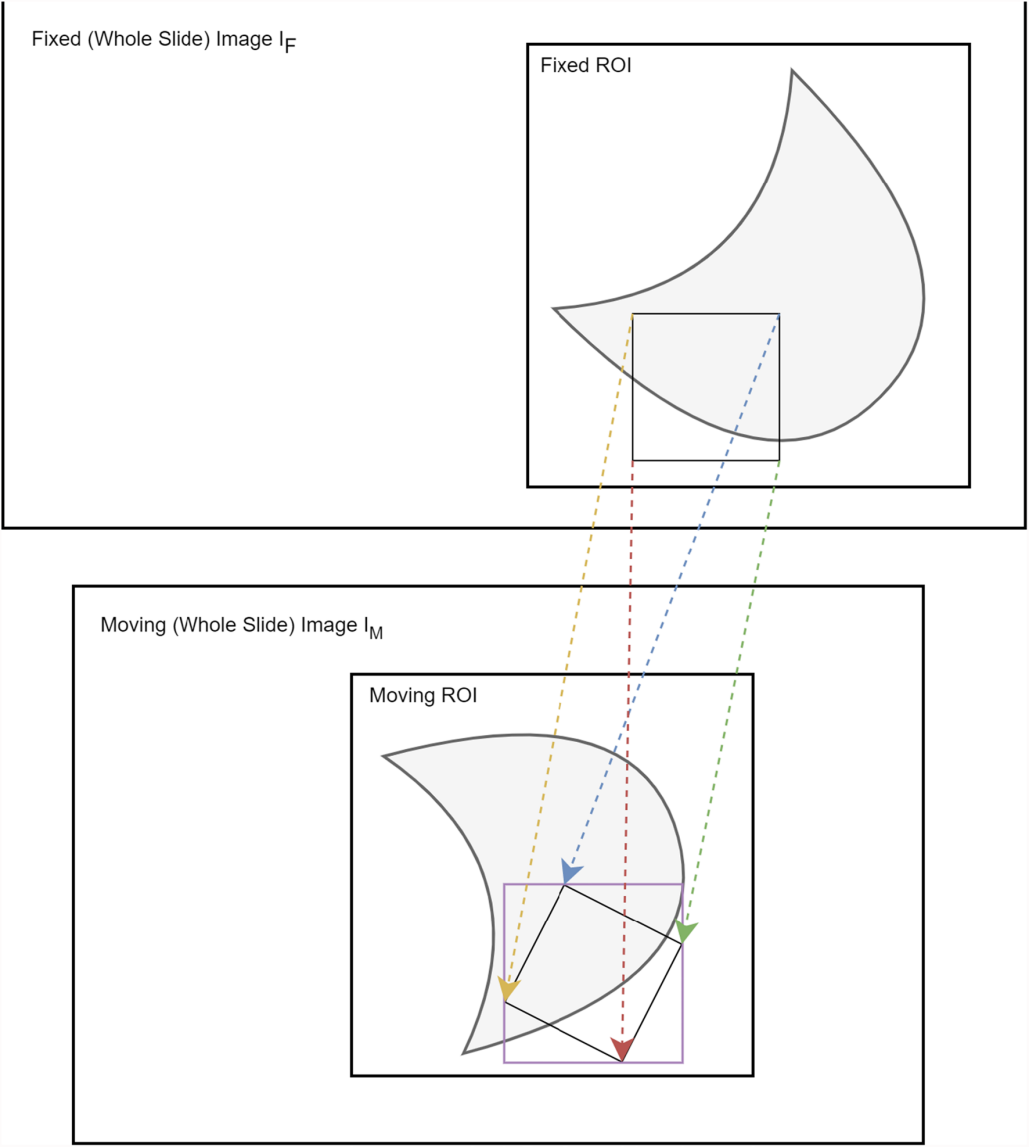
Transformation of a region of interest and its bounding box (purple rectangle) concerning the absolute coordinate system of the corresponding Whole Slide Images

Afterward, the calculated parameters need to be extrapolated to fit the dimensions of the base layer, and the previous pre-modifications (mapping to the ROI, large gap rotations or flips) have to be included in this transformation schema. The parameter map will serve as a base transformation for the additional and subsequent registration of detailed image structures or partial regions of the WSI. In detail, the preprocessed transformation parameters need to be applied to the upscaled and transformed coordinates of the moving image on the base layer, where particularly the initial rotation and translation differences are compensated. First, the region’s coordinates are translated in x and y directions to equalize the ROI’s absolute position on the WSI and are then rotated by the initially calculated rotation angle. Figure 4 sketches the idea of this procedure. On this occasion, the geometrical centers of our initial ROIs serve as the center of rotation.

This process results in two roughly equal regions on the reference and corresponding WSI that can be used for the subsequent refinement registration steps. However, the challenge lies in the fact that only rectangular, horizontally and vertically centered image tiles can be obtained from the data formats and processed using respective libraries. That means that the ROI’s rotation must be considered and thus needs to be wrapped in a bounding box to retrieve the extended image region from the slide image library. As this image section is subsequently used for registration, the image tile’s rotation has to be compensated and then cropped again to the size of the preceding ROIs’ dimension while using the geometric center of the image as the ROIs center of mass. In the following, we will present two strategies to enhance the coarse initial registration results. The drawbacks and advantages of these strategies will then be evaluated and discussed in the results section.

### Strategy I: clustered coordinates refinement registration

The first refinement approach focuses on the registration of large image sections to quickly transfer a potentially large number of spatially clustered annotations into a target coordinate system. Therefore, the fixed image is divided into equally sized image regions and will thus open up a grid depending on a) the number of sections or b) the fixed size of these grid tiles. The grid coordinates are then mapped, based on the previously calculated coarse transformation parameters, to the moving image coordinate system, resulting in a rough mapping between fixed (T_I) and moving tiles (T_M). Once again, the region must be extracted with its global dimensions from the moving WSI, taking into account scaling and rotations from previous transformations. However, this also means that the rotation parameter of the transformation has to be compensated and the image region cropped accordingly. The resulting image section is then used again as input to the subsequent refinement registration, serving as the moving image. The fixed image is the respective region of our previously rigidly created grid tile. Since these two image inputs are already well positioned due to the previous coarse alignment, the registration parameters can be strongly optimized, especially with regard to the number of optimization steps, sampling points or resolution levels, which greatly reduces the overall computation time. Nevertheless, this has to be decided on an individual basis since the result is strongly dependent on the absolute size of the image regions and the content included. With very large image sections, there may be discrepancies between the histological structures of the tissue, becoming even more pronounced in edge areas of the image regions, which is why the use of a non-rigid registration may be useful here. These increasing inaccuracies, amplifying from the image center to the corner regions, occur because the geometrical center of the image is used as the initial origin of the registration. For instance, using the upper left or the center of mass as the origin would result in similar effects at different parts of the transformed image.

The method allows for fast computation of a transformation rule to map a large number of coordinates from one image coordinate system into another. As long as these coordinates are mostly bound to a particular fixed region, the number of processable image tiles will be relatively low. Conversely, when annotations have been distributed homogeneously on the slide, the processing time might easily increase exponentially. In both cases, it is reasonable to determine certain image regions (e.g., region of interest) beforehand and pass them as input parameters to the registration algorithm.

### Strategy II: single coordinate refinement registration

As previously described, the transformation of homogeneously distributed annotations on the tissue can cause a considerable consumption of time and resources when using Strategy I. Therefore, we introduce a second approach dedicated to the accuracy of single annotations rather than large bulk transformations in a reasonable time. Thus, the initially coarse-transformed annotation coordinates are iterated, retrieving tiny image sections from the base layer (or, in the case of different magnification levels, the respective maximum resolution level) of both the fixed and moving WSI and performing a fast rigid registration. These small image patches are ideally squares and have a fixed width and height with the annotation coordinate as the center of the patch. We propose an edge length of about 128 to 256 pixels. These patch sizes were sufficient for our experiments in terms of accuracy and computation speed. As stated previously, a user might want to align two slides scanned at different magnification (e.g., 40× vs. 20x). In this case, we use the base layer of the slide with the lower resolution as the reference base layer and select the respective layer with an equivalent magnification level for registration. However, before retrieving the plain image data, the initial orientation of the WSIs must be taken into account here, as otherwise, an image section will be obtained that is far off the actual coordinate locations. In addition, the inverse rotation of the previously calculated coarse transformation parameters must be applied to the image section to include the rough registration in the further registration step since the WSI access utilities do not allow the retrieval of rotated image regions. Subsequently, the registration correction step can be performed, where only the displacement, rotation and similarity between fixed and moving images are computed (affine registration). The resulting transformation parameters are then added to the overall parameter list, serving as input for the transformation step.

### Transformation

The transformation of the coordinates is decoupled from the actual registration process, whereby the calculation of the transformation parameters can occur independently of applying the computed transformation to concrete coordinates. This allows to compute a comprehensive point-precise mapping without knowing the coordinates to be transformed in advance. Thus, differentially classified annotations (e.g., tumor or non-tumor) can be processed independently using the same transformation parameters without increasing the execution time for additional annotation classes. However, this scenario can only be realized with the first registration strategy.

The transformation parameters are successively applied to the individual coordinates in the same order in which they were created. The interpolation of the resulting continuous coordinates is performed after all displacement alterations are applied. To map the continuous points to discrete coordinates in the target coordinate system, we use a cubic interpolation with BSpline polynomials and a resample order of three [15].

### Limitations

A major limitation of this workflow is the high manual effort required when dealing with very heterogeneous datasets, which is inherently the case in digital microscopy. This is not only due to the manual processing route of slide preparation but also to the heterogeneity of the slide scanner landscape, scan profiles, and staining or tissue-related features. An important influencing factor is the geometric orientation of the tissue, both on the physical slide and within the digital data structure. Right angle rotations or mirrored image data are challenging to detect with conventional image processing algorithms and are already a hard-to-solve problem in their own domain. Further complications arise from the different magnification levels of the slides (e.g., 40× vs. 80x) or even visible discrepancies between slides with theoretically equal magnification levels, which is mainly due to the barely standardized nature of digital pathology. In addition to resolution-related and spatial influences, artifacts such as foreign objects on the slide, destroyed tissue such as tears, washed-out tissue or overlaps and digitization artifacts are further sources of error when automating the registration process. 

## Results

**Fig. 5:**
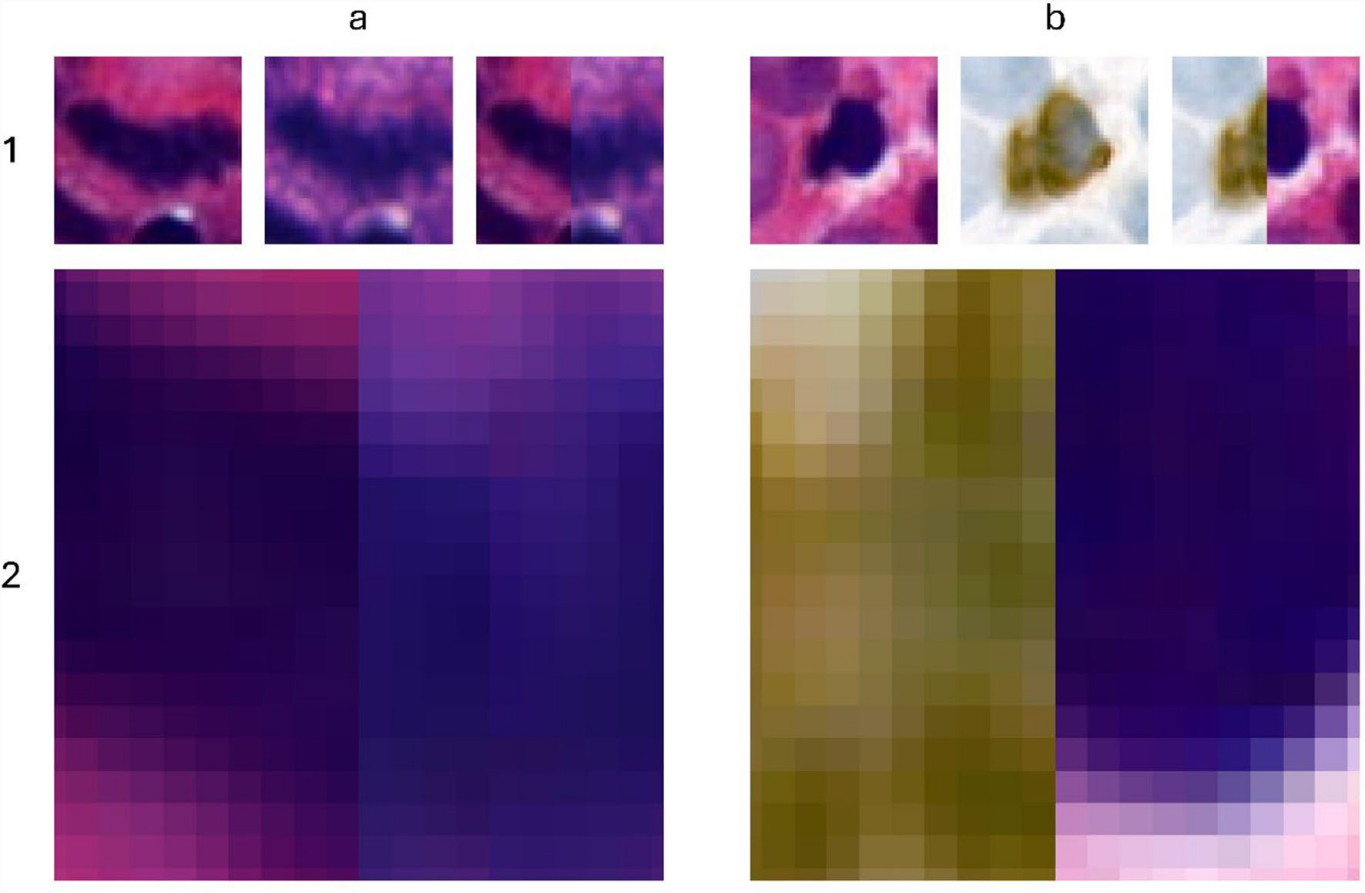
**a** H&E to H&E registration; **b** H&E to PHH3 registration; 1) Mitosis per scanner, comparing one half of each image; 2) Enlarged center of the combined images; These images highlight the challenge of providing pixel-precise annotations and segmentations. At lower resolution, the border between mitoses and stroma appears relatively distinct. At pixel level, however, it becomes impossible to perfectly distinguish between mitoses and stroma pixels.

The evaluation of registration algorithms is commonly performed using metrics to measure distances between fiducial markers and their average distribution around a mean or median error in variance, such as the target registration error (TRE) [18]. For the approach presented, such a procedure proves less meaningful in two different ways. First, the TRE (or similar metrics based on the correspondence of point pairs) refers to the totality of points transformed by a single parameter set. In patch-based approaches, this is similar in that multiple sets of points are each transformed by a single parameter set. Although the presented approach is also based on a patch-based registration, the final correction step gives each individual point coordinate a unique transformation rule. Second, our registration achieves such high accuracy that the corresponding points would have to be annotated with sub-pixel precision. Figure 5 shows why this is not even possible with the existing data.

It is clearly visible that the mitosis has a slightly different morphological shape depending on the scanner. This can be attributed to both the installed camera sensors/software (e.g., sampling, interpolation or color corrections) and the focal plane determined as sharped-edged by the scanning software. If two scanners capture the same mitosis at different points along the Z-axis, the mitosis will inevitably appear different, and a pixel-precise correspondence can no longer be achieved.

**Fig. 6:**
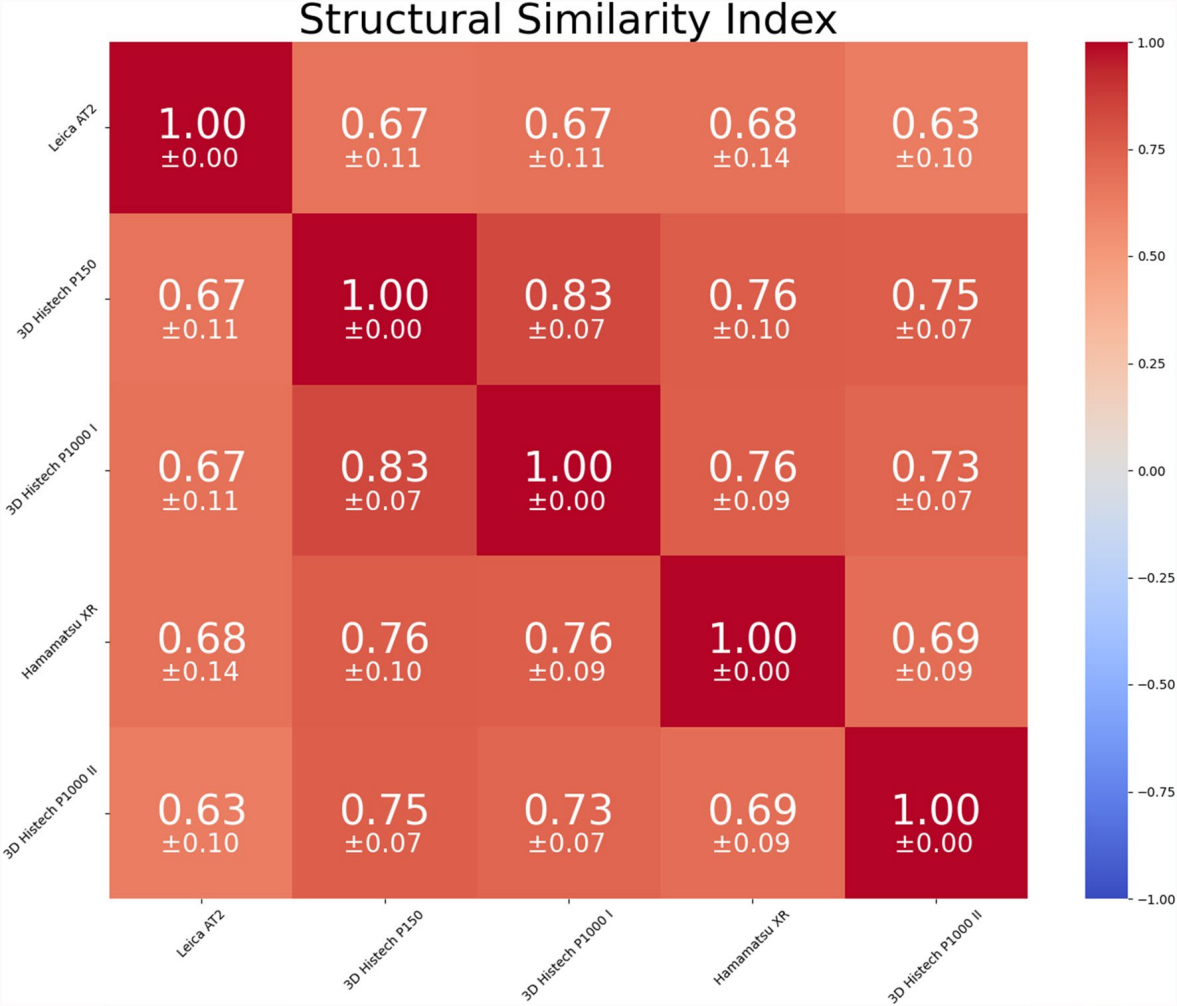
Overall SSIM between the five scanners assessed by colocalizing PHH3-based annotations to the five H&E WSI sets

Other metrics typically used are Jaccard Index (JI), Dice Coefficient (DC) and Hausdorff Distance (HD). JI and DC are based on the overlap of regions in the registered images, while HD represents the maximum distance between corresponding coordinates in two pointsets. All these metrics require accurate segmentation. However, as mentioned before, achieving such accurate, pixel-precise segmentation at the nuclei-scale is not feasible. To evaluate our algorithm performance, we registered all PHH3-based annotations (created on 100 slides and digitized with the 3D Histech P1000 II) to the corresponding H&E WSIs from each scanner and assessed the structural similarity index metric (SSIM) between the H&E regions. To minimize slide-based biases, we used the same number of annotations for each slide, based on the slides with the lowest number of annotations (n = 1). SSIM, which ranges from − 1 to 1, quantifies the similarity between two images. Values below 0 indicate dissimilarity, while values between 0 and 1 indicate similarity. For SSIM assessment, we created patches centered on the registered coordinates, rescaled them to the fit the patch dimensions of the scanner with the lowest resolution (3D Histech P150), and cropped the first and last rows and columns, accounting for uneven tile dimensions after resizing due to slight differences in resolution along the x- and y-axis in the 3D Histech P1000. The overall performance is shown in Fig. 6.

An in-depth analysis of the achieved SSIM scores was conducted to identify outliers and investigate their causes. For each annotation, we calculated the lowest SSIM for each scanner by comparing it with all the other scanners (details are provided in Appendix 1). The lowest observed SSIM was 0.31, which is in theory still interpreted as similar. However, 20% of the annotations fell below an SSIM of 0.5 and were visualized for further inspection (see Appendix 2). Using QuPath and manual measurement, the poorest results revealed offsets of approximately 33 μm (slide ID 32, SSIM 0.31), 20 μm (slide ID 37, SSIM 0.47), 9 μm (slide ID 1, SSIM 0.44) and 8 μm (slide ID 55, SSIM 0.42). In contrast, most other slides showed comparably lower or even negligible offsets. For cases with no offset, variations in tissue texture appearance or blurry image tiles were observed as contributing factors. The memory requirement and computation time are influenced by both the number of coordinates to be transformed and the distribution of these coordinates over the tissue. In scenarios with densely populated annotations, the previously introduced Strategy I aims to reduce execution time using fewer but, therefore, larger image tiles during the refinement step. Although this may introduce minimal distortion near the tile edges, it is a meaningful trade-off. Sparsely distributed annotations, with large distances between these annotations, on the contrary, indicate the use of Strategy II, which is tailored to using smaller tiles during the refinement step to circumvent unnecessary calculations. To illustrate the effectiveness of these strategies, we applied them to two WSIs of the same H&E-stained slide containing invasive breast cancer with sizes of approximately 28,000 × 46,000 pixels. For Strategy I, 100 annotations were placed within a compact area of 3500 × 3500 pixels, while for Strategy II, 100 annotations were placed across the entire tissue, covering an area of 12,000 × 20,000 pixels. The code was executed via Powershell on a system running Windows 10, with an Intel i7 CPU and 64 GB RAM. These experimental setups illustrate the nuanced considerations required for optimizing memory usage and calculation efficiency in diverse annotation scenarios. The resulting durations for the calculation using different parameters can be examined in Table 2.

**Table 2:** Overview of computation time for the refinement step performing registration of 10 densely packed (registration strategy I) or strongly dispersed (registration strategy II) annotations

Tile Size		1024 × 1024	2048 × 2048	4096 × 4096
Strategy I: dense annotations	Number of Tiles	19	8	4
	Number of Pixels	19.92 million	33.55 million	67.11 million
	Duration	55 min	21 min	14 min
Strategy II: sparse annotations	Number of Tiles	10	10	10
	Number of Pixels	10.46 million	41.94 million	167.77 million
	Duration	25 min	37 min	43 min

The computation time and memory requirement for the correction step depend solely on the number of annotations and chosen tile size. Consequently, the distribution of annotations within the tissue does not impact this particular step. While larger tile sizes necessitate more computation time per annotation, they facilitate higher accuracy by incorporating more mutual information. However, we would not recommend using tiles larger than 512 × 512 pixels to keep a balance between computational effort and actual added value. Table 3 provides the computation time per tile size during the correction step.

**Table 3:** Computation time for the correction step for differently sized image tiles

Correction	Tile Size	128 × 128	256 × 256	512 × 512
	Duration per Annotation	1.8 s	5 s	9.5 s

It is apparent that the tile size for the refinement step should be selected in accordance with the distribution of the annotations. With Strategy I, increasing the tile size leads to a significant increase in the total number of registered pixels, but the reduced overhead for each individual tile ultimately reduces the computation time by a factor of almost 4. In the case of Strategy II, a contrary effect can be observed. The larger the tile size selected and, therefore, the more pixels registered, the longer the total computation time. This is due to the fact that the annotations are so far apart that two individual annotations do not lie within the same image tile. An increase in the tile size therefore only results in more pixels being registered and thus increases the total computing time without adding additional value. The data shows that a tile size of 2048 × 2048 could not achieve the fastest computing time with either of the two strategies. However, this does not mean that there is no distribution of annotations for which this might be the case.

## Discussion

Depending on the staining of the tissue and the focal plane of the respective scanner, the acquired objects to be registered may show morphologically relevant differences [19]. The presented approach is robust to these deviations and can still achieve sub-micrometer level accuracy when aligning WSIs from different scanners or in different staining. However, this high accuracy is paid for with heavy memory allocation and protracted computation time since registration is performed on two hierarchical levels. First, a global registration is performed to get a rough alignment of the relevant image parts. This step is subsequently followed by a tile-based registration on the base layer of the WSI. Depending on the use case (e.g., high vs. low-density annotation distribution), this is achieved either by a coarse, rigid grid on the base layer or by very small individual image tiles at the respective annotation position on the WSI (see Strategy I vs. II). These two strategies can be regarded as two extremes when approaching the same registration problem. For larger datasets with significant computation time, we recommend investing some time in understanding how to configure the parameters concerning the specific dataset.

Before this, however, some pre-processing must be performed to compute a rough pre-registration, which is indispensable for using Elastix [1]. For this, aspects such as tissue segmentation or the rotation or mirroring of the respective scanner results must be taken into account. Pre-segmentation to leverage the accuracy of a coarse registration has proven challenging. On histological slides, smaller ablated tissue particles may be present in addition to the main tissue. Furthermore, parts of the tissue may be missing, or various artifacts may cause additional objects to be visible on the WSI of a section. Depending on the scanner, the WSIs may have varying content, which can be problematic for pre-registration because the segmented content is not congruent. In addition, if several slices have been combined on one physical slide, a manual visual inspection of the preregistration becomes necessary. This opens opportunities for further improvements, optimizing the workflow and minimizing manual effort.

It is possible to perform a complete registration of all the image tiles generated in the second step. This has the advantage that a global registration matrix can be pre-computed, and the transformation of the points can be done at a later time. If it is expected that multiple sets of points are to be registered between these WSIs, this can save time since the first two compute-intensive substeps only need to be performed once. However, since the last substep, where the respective points are registered again in their local neighborhood, is also quite time-consuming, this only leads to significant time improvement if a) different sets of points are registered and b) these are distributed over a larger area of the WSIs.

During H&E and subsequent IHC staining, we observed various forms of damage to the tissue. Sometimes, the tissue has completely detached from the slide, making the sections unusable afterward. In most cases, however, minor tears and deformations occurred. These can no longer be compensated for with affine transformations, so it seems reasonable to investigate further transformations with B-splines. Another source for potential variability within the image data can be found in the scanners. Several components in photoimaging devices, such as sensors or light sources, may degrade, reducing the color quality of the acquired pictures. However, recent studies showed that for digital pathology, these variances are still within an acceptable range [20].

While our high precision may make it difficult to give exact values, it can be shown quite well that it is significantly higher than the approaches previously described in the literature. However, such comparison might be considered inequitable, as these approaches are not designed to operate on the same re-stained tissue slides but rather on consecutive slides. The approaches presented in [3] and [4] achieve a deviation of 3.4 μm and 5 μm, respectively, in the best case. In the approach presented in [5], the accuracy is given as a percentage of the image diagonal, which, in the case of our sample WSI considered in the results section, would be 27 μm. However, if their algorithm performs equally well on a tile size of 128 × 128 pixels, they would achieve a deviation of 1 μm. Considering the smallest deviation of 3.4 μm for a WSI, this would correspond to 13.6 pixels for a WSI with a resolution of 0.25 mpp. A minority of our results performed worse than these numbers, the majority outperformed them. SSIM is typically used in radiology to evaluate registration improvement or to compare algorithm performance with other approaches [21–23]. In pathology, however, its use is rather limited [24, 25], with other metrics such as TRE, Dice Coefficient or Normalized Gradient Fields being more commonly employed. From our perspective, providing sufficiently accurate reference data for reliable algorithm performance assessment is not feasible. Instead, we outlined our approach to assessing the SSIM in a mutli-scanner, multi-stain registration problem and encourage others to adopt and incorporate SSIM in performance evaluations. Comparing the computation times between these algorithms and our solution is highly dependent on the slides and annotation distribution. The approaches outlined in [3] and [4] share similarities with our solution. In [3], various parameter sets are systematically evaluated to select the most efficient configuration, an aspect that could potentially be integrated into our setup. In [4], a two-level hierarchical approach using Elastix is employed, with non-linear transformations applied both for coarse registration and for patch-wise registration at higher resolution. If the same configuration were used in our approach, the computations would align with those reported in their paper. In [5], a significantly faster registration is reported. However, this algorithm, like the others, is designed to work on different tissue slides rather than optimized to work on the same but re-stained slides.

The novelty of our approach does not lie in the hierarchical use of Elastix but rather in its application to our specific use case. Our test data included two different stains, WSIs digitized with five different scanners per slide, located at three different university hospitals, with release dates spanning 2010 to 2018. All slides underwent quality control to exclude those with out-of-focus tumor regions. Further investigations into noise robustness regarding lab-specific H&E staining, other IHC markers with significantly differing DAB-to-Hematoxylin ratios, additional scanning devices and the impact of out-of-focus regions would provide deeper insights into our approach’s performance.

## Conclusion

The registration method presented here can achieve submicrometer accuracy, even in the presence of tissue deformations or staining- and scanner-induced morphological variability. This is achieved by a three-stage procedure that uses a global transformation, a subsequent registration of the individual WSI tiles and finally, another registration of the respective points based on their local neighborhood. This method can transform arbitrary point clouds between WSIs but is not intended to transform entire WSIs. However, the transformed points can be used to establish a correspondence between the WSIs, which then can be used as a basis for an image transformation. The high accuracy of this method is mainly achieved by immense computational power and duration, which can amount to several hours per WSI pair. Thus, the method is not necessarily the first choice when viewing multiple registered WSIs or when a fast operation is the main concern, thereby narrowing its applicability. However, when generating a ground truth for training AI algorithms, for example, neither a complete image transformation nor a fast execution is relevant, which is why this method is highly relevant in this context. 

## Data Availability

All data used in this study were generated under an inpatient treatment contract and are governed by the Berlin State Hospital Act (Berliner Landeskrankenhausgesetz). As such, the data are subject to strict confidentiality and data protection regulations and cannot be shared publicly. Access to the data requires prior approval from the Charité – Universitätsmedizin Berlin Ethics Committee.

## Appendix

### Appendix 1

Lowest Structual Similarity Index Metric (SSIM) from one scanner to the other scanners for each regarded annotation. Color Map is set to the range from 0 to 1 (instead of -1 to 1) for better interpretability.

### Appendix 2

Annotations with a SSIM of less than 0.50.

## Footnotes

1 github.com/openslide.
